# Challenges of neural interfaces for stroke motor rehabilitation

**DOI:** 10.3389/fnhum.2023.1070404

**Published:** 2023-09-18

**Authors:** Carmen Vidaurre, Nerea Irastorza-Landa, Andrea Sarasola-Sanz, Ainhoa Insausti-Delgado, Andreas M. Ray, Carlos Bibián, Florian Helmhold, Wala J. Mahmoud, Iñaki Ortego-Isasa, Eduardo López-Larraz, Héctor Lozano Peiteado, Ander Ramos-Murguialday

**Affiliations:** ^1^TECNALIA, Basque Research and Technology Alliance (BRTA), San Sebastian, Spain; ^2^Ikerbasque Science Foundation, Bilbao, Spain; ^3^Institute for Medical Psychology and Behavioral Neurobiology, University of Tübingen, Tübingen, Germany; ^4^Bitbrain, Zaragoza, Spain

**Keywords:** stroke, motor rehabilitation, neural interfaces, neurofeedback, rehabilitative technology

## Abstract

More than 85% of stroke survivors suffer from different degrees of disability for the rest of their lives. They will require support that can vary from occasional to full time assistance. These conditions are also associated to an enormous economic impact for their families and health care systems. Current rehabilitation treatments have limited efficacy and their long-term effect is controversial. Here we review different challenges related to the design and development of neural interfaces for rehabilitative purposes. We analyze current bibliographic evidence of the effect of neuro-feedback in functional motor rehabilitation of stroke patients. We highlight the potential of these systems to reconnect brain and muscles. We also describe all aspects that should be taken into account to restore motor control. Our aim with this work is to help researchers designing interfaces that demonstrate and validate neuromodulation strategies to enforce a contingent and functional neural linkage between the central and the peripheral nervous system. We thus give clues to design systems that can improve or/and re-activate neuroplastic mechanisms and open a new recovery window for stroke patients.

## 1. Introduction

Stroke is the leading cause of long-term disability worldwide. More than 85% of patients affected by a cerebrovascular accident suffer from functional deficits in motor control as consequence of the injury (Langhorne et al., 2011). Both the initial and long-term treatments for these conditions result in a substantial economic burden on the families and the healthcare system (Kolominsky-Rabas et al., 2006; Lee et al., 2010; Feigin et al., 2016). Within traditional rehabilitation strategies, physical therapy is the overall accepted and standard method of rehabilitation for stroke patients. An example of physical therapy includes movement constraint therapy (MCT; Taub et al., 1999), which consists of the physical restraint of the healthy limb, forcing the patient to use the paretic arm/hand. Although MCT has shown positive effects in chronic stroke with some residual movement along with other motor disorders (Taub et al., 1999), patients without residual movement one year after stroke did not display an improvement after MCT (Wolf et al., 2006). Thus, this approach might not be suitable for stroke patients with low Fugl-Meyer Assessment (FMA) scores and limited residual hand movement (Birbaumer et al., 2008). Furthermore, although intensive rehabilitation has recently shown recovery potential in chronic stroke (Ward et al., 2019), other traditional rehabilitation treatments did not show significant efficacy for functional recovery over the long-term (Kwakkel et al., 2004; Bell et al., 2015; Wu et al., 2016). Alternative therapies including peripheral actuators such as robotic exoskeletons (Lo et al., 2010) or electrical stimulators (Hsu et al., 2012) have shown great potential in the field of motor rehabilitation as they permit a repeatable and intensive proprioceptive stimulation of paralyzed limbs. However, bottom-up treatments using these technologies (i.e., passive robot-aided movements or open loop stimulation paradigms) do not necessarily contribute to regain motor function in severely affected patients (Prange et al., 2009) since explicit voluntary and contingent motor intention is not necessarily present during movement (Song et al., 2013).

These severely affected and chronic patients have, therefore, very limited treatment options and often remain severely disabled on the long-term (Byblow et al., 2015; Winters et al., 2015). Due to the aforementioned limitations of standard therapies, modern rehabilitation protocols for stroke have focused on the reactivation of the top-down pathways to restore the volley of voluntary contractions to the peripheral nervous system (Ramos-Murguialday et al., 2013). These therapies aim to assist the re-organization of neural circuits still intact following stroke by leveraging neuroplastic properties of the adult central nervous system, in order to convalesce motor function (Belda-Lois et al., 2011). These novel therapies are based on the use of neural interfaces that enable patients to employ their neural activity for controlling various rehabilitative neuroprostheses like robots or electrical stimulation devices and stimulate their peripheral nervous system in a contingent manner. Consequently, patients can receive feedback regarding their actual motor intentions or potentially adopted ineffective compensatory strategies, based on their remaining neurophysiological activity. Therefore, assuming that certain neural networks and pathways remain unaffected by the damaging effects of a stroke and can still transmit sensory signals to the brain and residual efferent motor commands, synapse-based learning facilitated by neural interfaces could assist in the restoration or development of alternative functional circuits following a stroke.

Therefore, our view for effective neural-interface-based stroke rehabilitation is the development of new top-down protocols based on reinforcing the functional link between the brain and the muscles. We focus on neural interfaces coupled with peripheral stimulation systems that provide naturalistic feedback to the patient. In order to describe our view on the topic, the remaining of the manuscript is divided into the description of neural interfaces (NI) with a major focus on brain-computer interfaces, a summary of some of the difficulties faced by researchers when combining neurophysiological activity sensing and peripheral stimulation technologies within a closed-loop system, some important aspects that should be considered when designing a NI for motor recovery after stroke and some scientific evidence correlating the use of NI to improvement of motor function and rehabilitation outcome. Finally, we provide a short discussion and conclusions.

## 2. Neural interfaces

Neural interfaces translate neural signals recorded from the central and/or peripheral nervous system into commands to allow users to control, for example, different types of rehabilitative feedback systems. Examples of the latter are robots or electrical stimulation devices that are used to activate paralyzed muscles. We consider these apparatuses “peripheral actuators” because they stimulate the peripheral nervous system. Other examples are brain stimulation devices (e.g., transcranial Magnetic or deep brain stimulation). These “central actuators” activate the perilesional or contralesional brain regions directly and could be defined as nervous system actuators.

**Figure 1:**
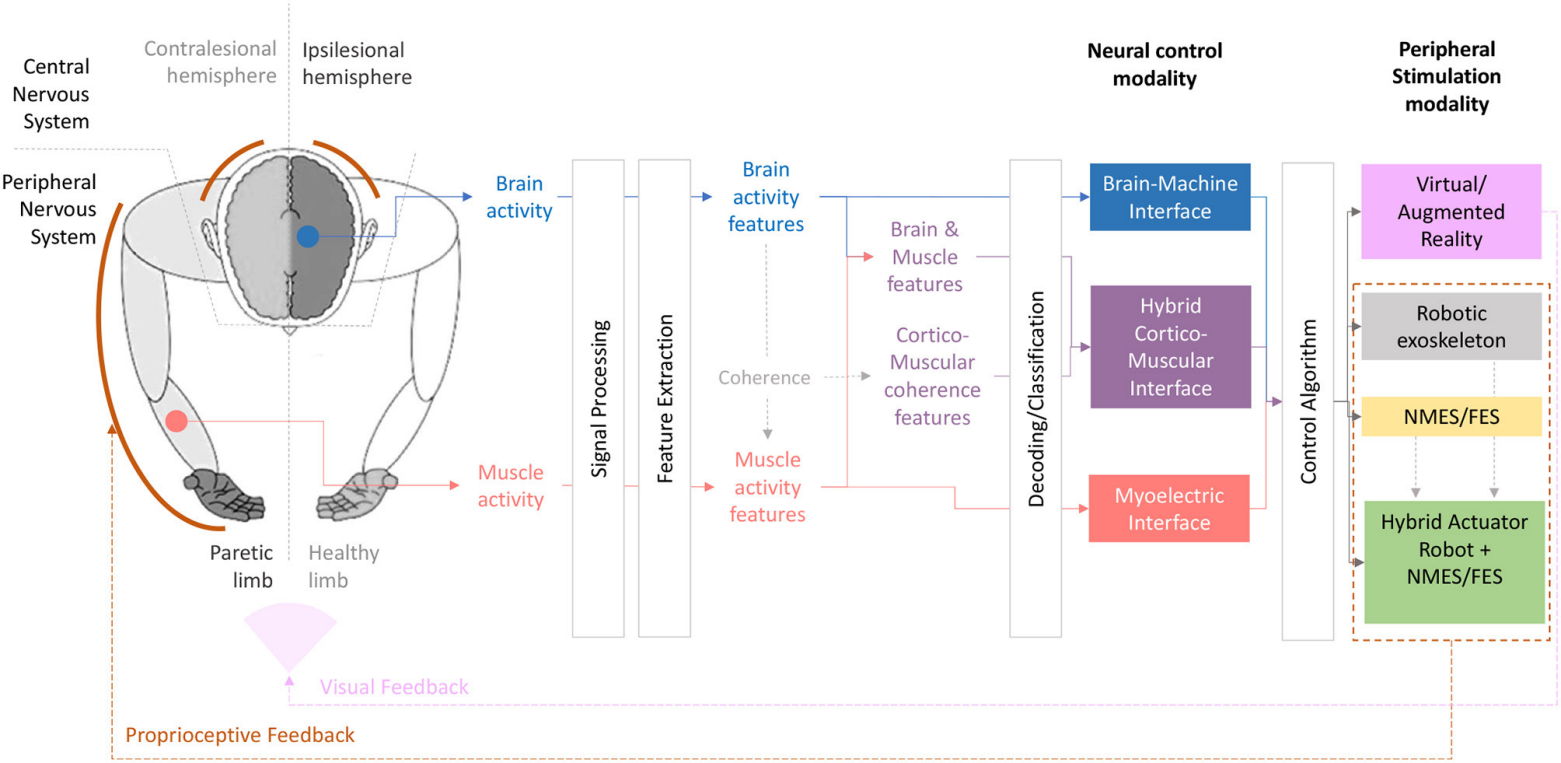
Visual summary of different neural control modalities coupled with different peripheral actuators proposed for upper-limb rehabilitation.

As a summary of the previous concepts, Figure 1 depicts an illustration of a neural interface to perform upper-limb motor rehabilitation.

Reading out the neural data in real time and using it (e.g., decoding motor intention) to produce an output signal that controls a nervous system actuator by bypassing the lesion is hypothesized to close the loop within the nervous system. In our case, it closes the loop between the movement intention and that exact feedback provided through the peripheral system. Closed-loop neural control systems allow interfacing with the nervous system by recording neural signals and simultaneously providing some type of neuro-feedback and/or stimulation to the nervous system. The motivation behind closed-loop approaches lies in the hypothesis that contingent proprioceptive feedback associated with the movement intention/attempt might activate plasticity mechanisms and strengthen the neural circuits, thereby promoting motor recovery. Thus, this interactive engagement with the nervous system can leverage Hebbian plasticity mechanisms inducing both long-term and short-term plastic changes (Fetz, 1969, 2007; Birbaumer, 1999) and ultimately lead to functional rehabilitation (Ramos-Murguialday et al., 2013). In the absence of any residual peripheral activity (i.e., muscle activity), the attempted/imagined movement of an affected limb in severely paralyzed patients can be decoded from brain signals. These signals provide an objective measurement to detect when and how patients try to move a paralized limb.

Indeed, brain activity of stroke patients has been employed to bypass the brain lesion and reinforce perilesional areas with the goal of reconnecting or optimizing the connection between brain and muscles. In this way, stroke patients can use their brain activity to control a virtual hand or different types of rehabilitative body actuators. In this last case, the precise association of cortical activation and peripheral feedback can boost neuroplasticity by re-establishing contingency between ipsilesional cortical activities, which is related to motor planning of attempted execution, and the proprioceptive feedback. The working hypothesis is that Brain-computer (BCIs) or Brain-machine Interfaces (BMIs) might strengthen the ipsilesional sensorimotor loop and foster neuroplasticity that facilitates motor recovery by triggering Heabbian plasticity (Birbaumer and Cohen, 2007; Jackson and Zimmermann, 2012; López-Larraz et al., 2018c). BCIs/BMIs record and process brain activity to obtain patterns that are subsequently decoded and used, for example, as a control signal in an online manner. In this work, and in relation to a tendency in the community to distinguish between both terms Wolpaw et al. (2020), technology using non-invasive brain signals is termed BCIs and invasive systems are BMIs.

Finally, the rehabilitation of patients with total or partial motor paralysis of one or more limbs by means of NIs has attracted considerable attention (Mateo et al., 2015; Pichiorri et al., 2015; Cervera et al., 2018; López-Larraz et al., 2018c; Mrachacz-Kersting et al., 2021) and these systems are the focus of intensive research due to their capacity to connect the human brain with external devices able to stimulate the nervous system in different ways (Blankertz et al., 2010; Millán et al., 2010; Chaudhary et al., 2016).

## 3. Design of brain-based neural interfaces for motor rehabilitation

Neural interface systems are usually composed of several modules, including the acquisition and processing of neural signals, a feature extraction and a classification/decoding module. Extracting features is a very important step in these systems because they contain characteristics of the brain activity that are informative for the interfaces and allow their classification. Also, how neurofeedback is provided to the user plays a crucial role. In the following we consider each of them in the framework of motor rehabilitation.

### 3.1. Dealing with noisy brain signals

The presence of artifacts and noise in brain signals is a main issue in EEG and a recurring research topic (Klass, 1995; Urigüen and Garcia-Zapirain, 2015). In signals acquired from stroke patients, possible noise sources are eye, head, or body movements during the attempt of a movement, muscular activity of the neck and cranial muscles, and also involuntary movements non-related to the task (López-Larraz et al., 2018a). Artifact removal is important to ensure that the features used are directly related to voluntary motor intentions because, although researchers always try to minimize the amount of artifacts during the recording, it is hard for patients to perform the task avoiding body and eye movements. In presence of artifacts, classifiers might artificially boost movement decoding accuracy due to noise related information (López-Larraz et al., 2018a). This implies the risk that patients might learn to control the BCI/BMI device generating artifactual signals. In fact, it has been shown that eye and head movements might pollute brain signals of patients to the extent that EEG motor correlates might be solely based on artifacts (Bibián et al., 2021).

There are two main approaches to deal with artifacts in closed loop systems: automatic trial rejection and/or algorithmic artifact correction (Winkler et al., 2011; Daly et al., 2014). Trial rejection is limited to training datasets containing a sufficient amount of data (Ofner et al., 2017). When trials cannot be discarded, signal correction is usually employed. Depending on the characteristics of the noise, different approaches can be considered.

For example, when artifacts are located in a frequency range other than the band conveying discriminative information related to motor attempt (i.e., alpha o beta bands), they can be easily removed using band-pass filters. For those cases in which the frequency band of the artifact overlaps with our band of interest, several denoising methods have been proposed, such as ICA or linear regression-based algorithms (Schlögl et al., 2007; Winkler et al., 2011; Daly et al., 2014). However, linear regression based methods are not able to remove non-linear artifacts.

Removing non-linear noise is important for NIs for motor rehabilitation because they are often coupled with neurostimulation techniques such as functional electrical stimulation (FES) or transcranial magnetic stimulation (TMS; Hoffmann et al., 2011; Vidaurre et al., 2013, 2019a, 2021; Rogasch et al., 2014, 2017; Iturrate et al., 2018; Kohli and Casson, 2020; Insausti-Delgado et al., 2021). Artifacts of short duration and high amplitude such as electrical stimulation pulses can be removed using median filters without substantially affecting the signals (Insausti-Delgado et al., 2021). However, recordings of brain activity are easily corrupted by electromagnetic fields that negatively affect the signal, masking the activity of interest (Walter et al., 2012). As aforementioned, this noise causes a biased closed-loop control that could cause maladaptive motor learning. Recently, Vidaurre et al. (2021) showed that eliminating spatial components at specific frequency bands could also reduce noise in other bands, in particular at the motor imagery frequency range. More recently, Chen et al. (2022) developed an algorithm to remove harmonic noise in different frequency components.

Regarding invasive recording technologies, such as electrocorticography (ECoG) and intracortically implanted microelectrodes, these are more robust to ocular (Ball et al., 2009) and EMG relaled artifacts (Freeman et al., 2003). However, they can also be corrupted by noise. Again, methods like narrow band-pass filters, ICA or stationary wavelet transform have been explored to identify and remove artifacts (Islam et al., 2014). Unfortunately, artifacts are usually difficult to identify given the highly non-stationary nature of these neural signals (Kaneko et al., 2007).

### 3.2. Voluntary and involuntary compensatory movement correlates

Although somehow related to signal noise, compensatory movements cannot be observed as such in the brain signals but have a huge influence in NIs for stroke rehabilitation. After a stroke, neural plasticity mechanisms activate to compensate the loss of motor function (Takeuchi and Izumi, 2012). Adopted neural strategies might contribute to motor recovery while others might actually limit it (Takeuchi et al., 2007). Compensatory movements are common after a stroke and brain patterns associated to them should be identified and isolated to ensure correct BMI/BCI feedback to the patient (Cirstea and Levin, 2000). The correlations between cortical activity and kinematics of joints that should not be involved in the intended movement were investigated and defined in Spüler et al. (2014). NIs for stroke motor rehabilitation should exclusively train the reinforcement of non-pathological neural patterns. Therefore, “healthy/functional” biomarkers should be used as biotargets. The usual example would be the reinforcement of ipsilesional activity to re-balance brain laterality (see cf. Ray et al., 2020).

### 3.3. Feature extraction module

Feature extraction is a crucial step in the design of BCIs/BMIs that allows extracting relevant information from brain activity (Blankertz et al., 2007; Krusienski et al., 2011; Bashashati et al., 2015; Ramos-Murguialday and Birbaumer, 2015).

The most prominent effects measured in non-invasive signals (electro or magnetoencephalography) are related to the modulation of power in different frequency bands. Regarding movement intention, these changes are termed event-related de/synchronization of sensorimotor rhythms (ERD/ERS; Pfurtscheller and Da Silva, 1999; Pfurtscheller and Neuper, 2006; Scherer and Vidaurre, 2018; Yao et al., 2018a). In fact, ERD/ERS in sensorimotor rhythms (SMR) were the first exploited features in EEG-based BCIs designed for stroke rehabilitation (Buch et al., 2008).

Regarding the extraction of features from brain signals of stroke patients, in general two distinct philosophies have been pursued. On the one hand, BCIs for motor rehabilitation of stroke patients might use a low number of electrodes located over the sensorimotor cortex in the ipsilesional hemisphere. The idea behind this approach is to minimize the time necessary to perform rehabilitation sessions by employing easy to use and fast to setup configurations of electrodes and other hardware and software. In BCIs, performance improvement is often achieved with the application of spatial filters that reduce volume conduction artifacts inherent to EEG data (van den Broek et al., 1998). Commonly used non-data driven spatial filters are bipolar, laplacian or common average references. These assign specific weights to the electrodes depending on their location (Ramoser et al., 2000; Tsuchimoto et al., 2021) and can be used with a low number of electrodes.

These simpler BCIs were used by chronic stroke patients with severe paresis. The patients were able to control a robotic orthosis fixed to the paretic limb using brain signals and decreased the power of the sensorimotor rhythm over the ipsilesional motor cortex measured with EEG (Ramos-Murguialday et al., 2009, 2012, 2013). Remarkably, functional motor improvements were stable in time as confirmed in a follow up measurement 6 months after the intervention (Ramos-Murguialday et al., 2019a). Patients showed a consistent pattern of brain reorganization and recovery. Theses findings have been also confirmed by other independent controlled studies (Ang et al., 2014; Pichiorri et al., 2015; Biasiucci et al., 2018; Cervera et al., 2018; López-Larraz et al., 2018c).

The second approach consists on searching the optimal algorithms that increase decoding accuracy to improve control by recording many electrodes. Acquiring signals from many sensors allows the use of data-driven approaches (Millán et al., 2010; Sannelli et al., 2011, 2016, 2019; Pedrocchi et al., 2013; Vidaurre et al., 2013, 2016, 2019a, 2021). Data-driven means that spatial filters are learnt from data depending on their objective function (Rivet et al., 2009; Pascual et al., 2011; Haufe et al., 2014a; Nakanishi et al., 2017; Jorajuŕıa et al., 2022) increasing thereby the discrimination and signal to noise ratio of the features (Ang et al., 2014, 2015; Ono et al., 2014; Antelis et al., 2016).

Within those techniques, there are supervised and unsupervised algorithms, depending whether or not they use information from the type of task. Regarding power changes, the most prominent supervised data-driven method to obtain spatial filters in BCI is usually termed Common Spatial Pattern (CSP) analysis (Ramoser et al., 2000; Blankertz et al., 2007). In short, it learns the weights of the electrodes such that the power difference between classes is maximized. This is optimal for exploiting ERD/ERS effects on EEG. Thus, it is a very successful and standard approach. Furthermore, the patterns obtained can be interpreted in terms of networks of sources, because they are in line with the linear model of the EEG (Haufe et al., 2014b). Hence, it is possible to locate them using methods such a scMUSIC or eLORETA (Pascual-Marqui et al., 2011; Shahbazi et al., 2015) and study from which sources discriminative brain activity arises. Thus, they can be helpful to determine the neural patterns of interest for closed-loop neurofeedback.

However, when the number of recorded electrodes is high, CSP has the drawback that high dimensional data covariance matrices must be estimated and often this estimation suffers from a strong bias (Blankertz et al., 2011). This is specially the case when the number of EEG samples acquired is low, which is common in calibration sessions with stroke patients in the clinical practice. Thus, many modifications have been proposed to improve their performance (Wang and Zheng, 2008; Kang et al., 2009; Lotte and Guan, 2010; Lu et al., 2010; Wang et al., 2011; Samek et al., 2012; Kawanabe et al., 2014). Another disadvantage of CSP filters is that they in principle need a relatively high number of electrodes to be computed, but these are not always available when working with patients data. This is because many electrodes imply a longer setup time. However, modifications have also been developed to use CSP with lower number of electrodes (Sannelli et al., 2016). Nevertheless it is a very robust technique that has been used in stroke rehabilitation. For example, it has been shown that using Common Spatial Patterns Analysis improves performance in comparison to employing only three electrodes over the ipsilesional motor cortex of severely paralyzed stroke patients (López-Larraz et al., 2017).

Other approaches related to power differences are based on Riemannian geometry due to the extensive use of covariance matrices, or in general, (semi-)positive definite matrices in BCI research. In the context of classification, it consists on a multidimensional extension of the unidimensional variance thresholding, as explained in the review (Congedo et al., 2017). Perhaps, the major disadvantage of this approach in the context of clinical applications is the lack of neurophysiological interpretation that otherwise spatial patterns can offer.

Features different from power changes in EEG have also been employed. For example, amplitude modulations that can be observed at very low frequencies. They are usually referred to as movement related slow cortical potentials (MRCP; Pfurtscheller and Aranibar, 1979). MRCP modulations have been measured in stroke patients, including persons suffering from severe paralysis (Niazi et al., 2011; Jiang et al., 2015; Yilmaz et al., 2015; Pereira et al., 2017; López-Larraz et al., 2018a).

In the case of invasive techniques to measure brain activity, ECoG and local field potentials (LFP) acquired from intracortical recordings have a broader spectral band (above 40 Hz, Staba et al., 2002) that can provide a greater information about arm and hand movement classification (Anderson et al., 2012; Pistohl et al., 2012; Yanagisawa et al., 2012). Although the major movement-related information might be encoded in high frequency bands of ECoG and LFP signals, low frequency components may also serve as a valid feature for online closed loop control (Milekovic et al., 2012). A wise selection of most informative spectral power features (e.g., selected via a screening task; Leuthardt et al., 2004) and the combination of features extracted from different frequency bands might be the most optimal solution for improving invasive BMI control accuracy (Milekovic et al., 2012). Additionally, motor-cortical single (SUA) and multi-unit (MUA) spiking activity have been shown to encode both upper limb speed and direction information (Moran and Schwartz, 1999). Usually, models based on linear regression-like algorithms have been used for decoding upper limb motor intentions directly from the spiking firing rates of neuronal units or ensembles for controlling virtual arm models (Ajiboye et al., 2017), high-dimensional robotic arms (Hochberg et al., 2012; Collinger et al., 2013) or functional electrical stimulation (Bouton et al., 2016; Ajiboye et al., 2017) systems in individuals with intact cortical structures and more recently in stroke patients (Ramos-Murguialday et al., 2019b). For a review on this topic, please refer to Waldert et al. (2009).

### 3.4. Classification/decoding module

Although many classifiers are suitable for BCI applications (Lotte et al., 2018), BCI systems are still inefficient (Blankertz and Vidaurre, 2009; Vidaurre et al., 2011a,b; Sannelli et al., 2019). This problem could not be completely overcome yet (Nierhaus et al., 2021). And it is even more so in the context of rehabilitation and other clinical applications (Birbaumer et al., 2009; Lee et al., 2019). Modern neurofeedback systems include for example regression paradigms (Wu et al., 2017; Vidaurre et al., 2022), but nowadays, clinical settings call for simple classifiers such as linear models. These uncomplex models offer two main advantages: first, they usually need less data to be trained. This process is usually called calibration, a bottleneck in clinical BCIs (Lotte, 2015) because it is time consuming and a source of frustration for the patient; second, they are easier to interpret (Müller et al., 2017). Linear classifiers can be as simple as thresholds that are used to positively reward the patient when they produce the discriminative brain activity (Ramos-Murguialday et al., 2013; Pichiorri et al., 2015).

An important aspect that should be considered in NIs for stroke motor rehabilitation, is that neurostimulation is often used, and it interacts with the human body inducing the excitation of different nervous systems (Nitsche and Paulus, 2000; Ridding et al., 2001; Peinemann et al., 2004; Zrenner et al., 2018). The excitatory mechanisms affecting the neurophysiological signal that controls the closed-loop system might change the characteristics of the signals of interest. Therefore, classifiers adjusted by neurophysiological activity during a BCI/BMI intervention have shown to enhance motor performance in many different studies (Millán et al., 2007; Vidaurre et al., 2007a, 2013, 2019a; Blankertz and Vidaurre, 2009; Sannelli et al., 2011; Faller et al., 2012; Scherer et al., 2018; Spüler et al., 2018; Nierhaus et al., 2021).

### 3.5. Calibration and (co-)adaptation

Reducing or eliminating calibration periods is an active research topic (Lotte, 2015). A way to reduce calibration recordings is to employ data from the same subjects in previously recorded sessions (Vidaurre et al., 2007a), a strategy also tried in patients data (López-Larraz et al., 2018b). In the same line, in the first decade of 2000 it was shown that adding some data from the current day to past data to recalibrate the BCI improves performance (Vidaurre et al., 2007a; Azab et al., 2019), recently this was also tested in patients data (López-Larraz et al., 2018b). Other approaches include unsupervised adaptation between sessions (Arvaneh et al., 2013).

Another way to reduce or even eliminate calibration without feedback, is to perform online co-adaptation by incorporating user data to the feature extraction and classification modules trial by trial. Recently, it was shown in healthy participants that co-adaptation induces brain activity changes after just one hour of BCI training (Nierhaus et al., 2021). Previously, co-adaptation also proofed to be a useful tool (Vidaurre et al., 2005, 2006, 2007b, 2010; Yoon et al., 2009; Llera et al., 2011; Faller et al., 2012; Wu and Ge, 2013) that can reduce BCI inefficiency (Blankertz and Vidaurre, 2009; Vidaurre and Blankertz, 2010; Vidaurre et al., 2011a,b), a concept first introduced in Vidaurre et al. (2011a).

In the case of BMIs, online co-adaptation has been also proposed for intracortical recordings. It consists on updating part of the decoder based on a Kalman Filter using blocks of 1–2 min of data (Orsborn et al., 2012; Dangi et al., 2013) to adapt the initial decoder and improve performance.

As mentioned above, such recalibration and adaptation algorithms could be particularly useful in clinical applications, where many factors can limit initial performance and thus patient adherence to the treatment as recently shown in Zhang et al. (2022). Nevertheless, in clinical applications the level of algorithm recalibration or adaptation should be critically studied to only account for the non-stationarities of the neural signals and maximize the desired neural adaptation toward a theoretically stable functional map by means of cortical plasticity by the patient.

### 3.6. Sensory feedback modalities

Multiple sensory modalities are often used to assess the state of the body in reference to the external environment and are thus key for normal motor control (Rossetti et al., 1995; Van Beers et al., 1999; Sober and Sabes, 2005). They can be used as afferent information to feedback patients their current condition. In this section we review the aspects that we consider the most important in the design of neuro-feedback strategies.

#### 3.6.1. Proprioceptive and tactile feedback

The ability to correct errors in real time (part of the error-based learning) has been found highly dependent on the proprioceptive system (Gordon et al., 1995). Proprioception is also important for forming and helping to update a template of appropriate velocity-based motor commands for successful execution of a motor skill (Thoroughman and Shadmehr, 1999; Hwang et al., 2006).

Central processing of proprioceptive feedback is necessary for motor learning. Thus, stroke-related damage to somatosensory cortical areas, thalamus and/or the associated white matter tracts would result in impaired continuous motor learning (mainly affecting efferent pathways). For example, in Vidoni and Boyd (2008) individuals with chronic stroke of the middle cerebral artery were recruited and trained to perform a continuous motor learning task under severely restricted visual feedback. The aim was to investigate the role of proprioception in motor learning. Generally, some stroke subjects were able to demonstrate a behavioral change and thus show learning of the practiced pattern of continuous movements. Importantly, they found that proprioception was strongly related to the magnitude of behavioral change associated with learning. It could then be concluded that the integrity of the sensory processing system is the main predictor of the success of motor skill acquisition in stroke rehabilitation. The most relevant areas were somatosensory cortices, thalamus and the associated white matter tracts. When these sensory-associated regions are dysfunctional either due to an ischemic insult or transiently using TMS, learning is compromised (Della-Maggiore et al., 2004). If these structures remain intact, they are available to create representations of behavior through intra-cortical interaction even if one or more sources of feedback are disrupted. Most stroke patients exhibit relatively intact afferent tracts that should be engaged to drive the sensory information of a movement of the paretic limb to the brain. In this line, the integrity of somatosensory components of the impaired limb (e.g., skin mechanoreceptors, muscle spindles, Golgi tendon organs and joint receptors), responsible for transmission of afferent information, also conditions the benefits of the rehabilitation.

Consequently, proprioceptive feedback provided by neural interfaces can play an essential role on the rehabilitative effects, which directly depend on the remaining afferent pathways. Neural interfacing systems can act providing a contingent connection between efferent activation and afferent feedback (via vision or proprioception; Daly and Wolpaw, 2008; Chaudhary et al., 2016). And in fact, researchers have shown that somatosensory and proprioceptive feedback in the scope of NI are essential for motor control and learning (Ramos-Murguialday et al., 2012).

Furthermore, continuous feedback associated with cortical activity enhances the control of body actuators and potentiates instrumental learning, which might also favor Hebbian neuroplastic mechanisms (Jackson and Zimmermann, 2012; Ramos-Murguialday et al., 2013; Mrachacz-Kersting et al., 2016). Indeed, the afferent recruitment of somatosensory components due to a robotic orthosis or neurostimulators, generates changes in sensorimotor cortical rhythms of motor areas close to the lesion (Ramos-Murguialday et al., 2013). These changes are expressed as down regulation of inhibitory processes that result in cortical plasticity and reorganization (Golaszewski, 2017). However, research has shown that the efficacy of neurostimulation-based therapy crucially depends on delivering the stimulation while the patient is trying to perform the movement (Barsi et al., 2008). Although robotic exoskeletons can reproduce the desired trajectory or movement better, FES can activate muscles electrically, which are the ultimate output of the neuromotor system. Probably the simultenous use of orthoses/robots coupled with FES might be the most successful option, although their effect on brain oscillatory activity differs (Cho et al., 2023).

If we focus on using as feedback electrical stimulation to induce the movement, we know that FES seems (besides the artifact bias) to improve classification performance regardless whether it is applied over or below the motor threshold (Vidaurre et al., 2013, 2019a; Corbet et al., 2018). It also increases functional connectivity of sensorimotor areas in healthy subjects and in patients where it additionally relates to a motor deficit decrease (Mottaz et al., 2018; Vidaurre et al., 2019a).

The neural control of FES has been demonstrated on individuals with motor disorders (Daly et al., 2009; Young et al., 2014; Osuagwu et al., 2016; Ajiboye et al., 2017; Ibáñez et al., 2017; Trincado-Alonso et al., 2018). Further, controlled studies have been conducted to study if these interventions can overcome other approaches in stroke patients, such as conventional therapy (Kim et al., 2016), FES without BCI/BMI (Li et al., 2014; Mukaino et al., 2014), or FES triggered by a sham BCI/BMI (Biasiucci et al., 2018). In the four studies, triggering the FES with the movement intentions decoded with EEG led to higher recovery than the control interventions. In the latter study they also demonstrated that moderately-to-severely disabled stroke patients can achieve clinically relevant improvements and these can last at least 6 months after concluding the therapy (Biasiucci et al., 2018).

Another successful option to activate the sensorimotor afferents is tactile stimulation. Several works have also shown that it can improve the accuracy of motor attempt/imagination when performing BCI experiments. Notable examples are the studies presented in Yao et al. (2017), Shu et al. (2018), Yao et al. (2018b), and Zhong et al. (2022), showing the advantage of including tactile sensation concurrent to the performance of motor tasks.

Our advice to researchers would be that before running any neurofeedback-based intervention, measuring and reporting proprioceptive and haptic ability should be considered, as well as the location of the injury or severity of the insult and its functional effect on the proprioceptive system. If the sensory afferent pathway is disabled (inability to process the upcoming sensory information, which prevents “closing the loop”), afferent assessment might be an exclusion criterion for participation or at least for categorization in proprioceptive neurofeedback-based rehabilitative interventions (López-Larraz et al., 2018c).

#### 3.6.2. Immersive and multisensory feedback

Active involvement of patients during the rehabilitation procedure is also key to improved training results (Blank et al., 2014). Thus, rehabilitation schemes that involve multisensory feedback might have the potential to improve current outcomes. Examples of those are systems combining robot-driven actuations and electric or magnetic stimulation, immersive visual and auditory feedback by, e.g., embedding the training in a virtual world, or tactile stimulation (Hu et al., 2015; Resqúın et al., 2016; Straudi et al., 2016; Nam et al., 2017; Zhong et al., 2022).

Regarding immersive feedback, growing evidence shows that virtual reality (VR) and interactive procedures might be very beneficial for stroke rehabilitation (Yates et al., 2016; Laver et al., 2017). For example, the studies by Vourvopoulos et al. (2019a,b) found in one and four chronic stroke patients respectively, that the combination of EEG and VR is a safe rehabilitation protocol, that can induce neuroplastic changes. Furthermore, recently Sebastián-Romagosa et al. (2020) showed in 51 stroke survivors ranging from severe to mild impairment, that a BCI treatment also combining EEG and VR promoted long lasting functional increase of the upper extremity. Finally, a very interesting work combining transcranial stimulation with EEG and VR demonstrated in three stroke patients, that ipsilesional motor activity and behavioral function can be increased with this combination of techniques (Johnson et al., 2018).

In any case, VR together with other approaches such as robot-assisted proprioceptive feedback or transcranial stimulation, are promising research directions (Levin et al., 2015; Johnson et al., 2018; Zheng J. et al., 2018) and it is likely that future therapies will be based on systems using a combination of virtual reality gaming and proprioceptive feedback (Mattia et al., 2020).

### 3.7. Physiotherapy to generalize and exploit the rehabilitative effect

According to Ramos-Murguialday et al. (2013), although plastic changes taking place after BCI/BMI interventions might not automatically translate to meaningful activities of daily living, a physiotherapy training immediately after the BCI/BMI intervention could produce significant changes in motor impairment and it is thus highly recommended.

This shows that in any rehabilitation paradigm aiming to improve the quality of life of stroke patients, it is of great importance to include the individual motor goals of the participants and personalize the intervention to meet those goals. Thus, in order to harvest the best possible outcomes from the intervention, the BCI/BMI session should be embedded in a training program allowing personalization and the application of meaningful and functional movements.

Repeated training of functional movements including grasping a toothpaste tube, eating, reaching and grasping while standing and with social distractions has the advantage of representing natural movements which the patient could practice at home, maximizing the possibility of repetition and keeping the participants motivated and interested. Developing rehabilitation scenarios and interventions with such tasks is key for the successful generalization of any rehabilitation therapy, especially the ones using novel technology. Therefore, the application of a physiotherapy paradigm, which is both daily life oriented and goal directed, is highly recommended. The patient must be coached to achieve autonomy in exercises. Patients should also be encouraged to adapt behavior and to use the newly learned skills in daily life.

### 3.8. Mental and physical state

The increasing complexity of rehabilitation environments that include, for example, brain-controlled exoskeletons, electrical stimulation, auditory cues, and virtual reality might also have detrimental effects on the patients' ability to perform because of divided attention and cognitive deficits present after stroke. For example, the combination of a movement task and a cognitive task (proper oral response to specific auditory stimuli in a dual-task paradigm) showed a decrease in walking speed (Bowen et al., 2001). Effects on performance of upper-limb movements have also been discovered in a similar dual-task paradigm (Pohl et al., 2011). Thus, it is possible that decoding the mental state of the user of BCI/BMI systems might provide additional information to improve the rehabilitation outcomes. Furthermore, the learning experience of the patient could be enriched by adapting the task to the current mental state.

For example, Walter et al. (2017) developed an adaptive system for learning arithmetic that optimizes the task difficulty on the learner's current workload online. The work demonstrates that real-time measurement of cognitive load is feasible. This framework could be exploited in neural rehabilitation training settings to reduce task difficulty; however, including the mental state of the patient in the NI is currently not a well-established research topic.

Regarding published literature on the general population, Hogervorst et al. (2014) review and compare different methods for the electrophysiological assessment of mental workload. They conclude that EEG-based methods perform best, at least for the memory training tasks that were tested. Alternatively, EOG has also been used together with EEG to more reliable estimate by the user's workload or fatigue (Novak et al., 2015). Finally, Aricò et al. (2018) recently reviewed passive BCI systems that have been tested outside of the laboratory and provide an overview of different methods and potential applications.

In one of the few works on stroke patients, Park et al. (2014) explored how cognitive engagement could be measured to increase the outcome of motor rehabilitation. They attributed differences in the EEG activity of active and passive hand movements in chronic stroke patients to increased cognitive engagement during the active task. Besides, other potential neurophysiological correlates of cognitive workload during robot-assisted tasks have been discovered in Fels et al. (2015). In that work the authors could predict perceived workload of participants from interhemispheric network measures during resting-state EEG recorded prior to the rehabilitation training.

Not only workload, but also mental or physical fatigue, and attention or vigilance might have an influence on task performance. There is extensive research, especially focusing on drivers in the transportation sector, cf. Balandong et al. (2018) for a recent review. Roy et al. (2013) tried to disentangle working memory load and time-on-task. They claimed that especially the spectral power of lower alpha oscillations in the midline electrodes increased with mental fatigue. However, they acknowledged that other factors such as arousal might influence or overshadow this measure of fatigue. Particularly, tasks related to movement might be prone to that effect, since central alpha activity also represents a marker of movement (Pfurtscheller and Da Silva, 1999).

Event-locked measures of vigilance such as error-related-potentials could provide a marker that is more robustly discernible from the movement-related spectral changes. For example, Omedes et al. (2018) showed that error-related potentials can be used to detect erroneously decoded movement commands. These could, for instance, be employed in an oddball paradigm to measure vigilance of patients to the task.

Physical fatigue, particularly of muscles involved in the training exercises, might play a detrimental role in the decoding accuracy in rehabilitation environments including electromyographic control. Different methods for assessment of muscle fatigue using EMG have been reviewed (González-Izal et al., 2012). A first step toward evaluation of muscle fatigue in stroke patients undergoing neurorehabilitation has also been done (Ray et al., 2019). All these works indicate that, in order to optimize any rehabilitation intervention, neurophysiological inputs can be used to track different user states directly influencing rehabilitation (attention, cognitive load, fatigue etc.) that can then be used to adjust the system and the intervention on demand allowing longer and more efficient training sessions (Thacham Poyil et al., 2020).

### 3.9. User involvement and acceptance

It has been shown that acceptance, adoption, motivation, engagement and participation of patients are essential to therapy success (World Health Organization, 2003). Thus, the involvement of patients during the rehabilitation procedure is key to improved training results (Blank et al., 2014). In fact, the design of neural interfaces for rehabilitation includes decisions about their features that should ensure, not only the fulfillment of technical requirements, but also the acceptance of the final users. This means, that not only the neural-motor considerations should be assessed, but also feedback from the patients should be gathered and taken into account into the design. However, according to a recent review (Baniqued et al., 2021), studies on the topic hardly ever disclose information about the usability of the systems for motor rehabilitation, nor about the degree of acceptability of the final users.

A notable exception is the very recent study on the acceptance of BCI systems for stroke rehabilitation (Grevet et al., 2023), that has provided important guidelines for researchers. They stress the importance of informing patients about the goal of the BCI-based therapy, clarifying how it works and which can be the expected outcomes. They also remark that informing the users about difficulties that they might encounter, e.g., learning and cognitive costs, is also vital. In summary, a similar concept to the Goal Attainment Scale is highly relevant for the BCI/BMI field.

Another interesting recommendation found in Grevet et al. (2023), is that one way to let possible users clearly understand how a BCI system for stroke rehabilitation works is the production of videos. Pedagogical material for the general public can greatly benefit the perception of these systems. In fact, not only possible users should be informed, but also their relatives and care-givers.

Finally, the most important conclusion is that the experimental protocol should be adapted to the assessed acceptability so that the wellbeing and the engagement of patients can be optimized, which most surely will be translated into an increase of the efficiency of the rehabilitation therapy.

## 4. Biotargets for rehabilitative neural interfaces

Brain correlates associated with motor attempt or execution (e.g., ERD/ERS in sensorimotor rhythms) have been commonly used as the feedback feature or bio-target in BCIs for motor rehabilitation (Ramos-Murguialday et al., 2013). Neural recordings from recent clinical studies using NIs in stroke patients undergoing motor recovery have permitted the finding of neurophysiological correlates of functional motor improvement in brain and muscular signals. Hence, in this section we review works identifying brain and cortico-muscular features correlated to motor recovery in stroke that could be proposed as neural targets that might boost the rehabilitation outcomes of NI-based treatments.

### 4.1. Brain correlates

A few reviews have investigated how electromagnetic signals, as those used in BCI/BMI research, are correlated with upper-limb impairment or can predict the rehabilitation outcome in stroke (Finnigan and van Putten, 2013; Rabiller et al., 2015; Triccas et al., 2019). The amount of research in this area is increasing with the number of published clinical trials using a rehabilitation therapy based on neural control and the correlation of electromagnetic signals with behavior. The identified correlates are Sensory Evoked Potentials (SEPs), oscillatory cortical signals, measures of functional connectivity and measures of interhemispheric balance of brain activity (Triccas et al., 2019).

The work of Ramos-Murguialday et al. (2013) was among the first to report a change of laterality of brain activation related to motor improvement. The trial focused on chronically severely impaired stroke patients and correlates were discovered using functional MRI. Also, Mane et al. (2019) found that a brain symmetry index based on EEG power is the best predictor of gains in upper limb motor function for an intervention based on a brain-controlled exoskeleton. In a trial on a similar patient cohort an increase of brain desynchronization was found on both hemispheres between the pre and post measurement also accompanying recovery (Ono et al., 2014). Pichiorri et al. (2015) found increased desynchronization in the experimental group using a brain-controlled feedback paradigm, too. Furthermore, they also investigated coherence measures and found a correlation between the motor improvement and a weighted density measure in the beta band of the EEG. Other works have more recently also reported similar findings (Biasiucci et al., 2018; Carino-Escobar et al., 2019).

The strengthening of the ipsilesional motor pathway has been seen to cause anatomical and structural changes in the integrity of the descending cerebro-spinal tract. The case study report published by Zich et al. (2017) using motor imagery neurofeedback, training showed that the patient who benefited the most from the intervention had a significant increase in lateralization during motor imagery and attempted movement toward the ipsilesional hemisphere rather than to the contralesional one (laterality was reversed). The increased lateralization was attributable to be an increase in ipsilesional and a decrease contralesional activity. Similar findings were reported by Song et al. (2014). Increased ipsilesional cortical activation measured by fMRI (Kimberley et al., 2004; von Lewinski et al., 2009) was also found as an effect of electromyography-triggered electrical stimulation protocols. Similarly, the analysis of Diffusion Tensor Imaging data from Ramos-Murguialday et al. (2013) corroborates that the reorganization of structural and functional connectivity within the motor networks of the brain that occurs during the partial recovery of upper limb motor functions in severely paralyzed stroke patients was a result of a BCI intervention. The study findings provide evidence that the BCI, through reinforcing brain activity on the same side as the lesion and improving proprioceptive function in the affected hand, triggers changes in the connections and circuits involved in somatosensory and motor functions, including both within the hemisphere and between hemispheres. These alterations in the neural pathways associated with afferent and efferent connections support the partial restoration of the original motor control physiology, even in cases of severe and chronic strokes, as demonstrated in Caria et al. (2020). These results lead to the conclusion that interventions should aim at modulating features that entail increased ipsilesional and/or reduced contralesional activation.

Finally, the recent work Ray et al. (2020) showed in an observational study with 30 stroke patients that a progressive shift of alpha ERD toward the ipsilesional hemisphere correlates significantly with clinical improvement regardless of lesion location. Also, initial alpha ERD might be a key factor to stratify stroke patients, with its interhemispheric balance being determinant for motor recovery.

### 4.2. Cortico-muscular correlates

Some researchers have already explored hybrid BCIs that combine features derived from brain and muscle signals in the control paradigm (Leeb et al., 2011). From the technical point of view, supporting signals from muscles involved in motor intent have been employed to increase the performance of BCI systems (Leeb et al., 2011; Lalitharatne et al., 2013; Balasubramanian et al., 2018; Lopez-Larraz et al., 2018; Spüler et al., 2018) combining brain and muscle signals in different ways. Usually, the final classification decision is a weighted mixture of the decoding results of both modalities (Sarasola-Sanz et al., 2017; Spüler et al., 2018; Tryon et al., 2019). And actually, the aggregation of multiple decoding outputs is a usual technique employed in machine learning to fuse information of different decoders and obtain a single decision output (Fumanal-Idocin et al., 2021a,b, 2022).

Nevertheless, the combination of classification outputs is unfortunately not directly related to the communication between brain and muscles: on the one hand, brain features alone cannot ensure a functional connection to the movement due to their lack of specificity to the motor output, especially in non-invasive systems. On the other hand, the origin of motor signals is not only cortical, but they are generated in the spinal cord as well (Baker, 2007). Furthermore, there are several other sources of disagreement between brain and muscle signals that significantly increase the difficulty to detect movement intent (Balasubramanian et al., 2018). The patients brain is usually damaged, causing altered neural dynamics, because the sources contributing to functional motor control might also be located in (originally) non-motor brain areas (Johansen-Berg et al., 2002; Jang et al., 2005; Misawa et al., 2008). Additionally, often patients produce uncontrolled movements that greatly increase the amount of noise in the brain and muscle signals. Thus, combining both modalities (brain and muscle activity) has proven to be highly non-trivial.

In order to partially ameliorate these difficulties, over the past few years researchers in the field of stroke rehabilitation have started to focus on the simultaneous study of synchronized cortical and muscle activity to gain insights regarding the disrupted efferent mechanisms after a cerebrovascular accident. Methodologically, there are different types of synchronization or coupling between two signals that can be estimated: phase-phase, amplitude-amplitude, and phase-amplitude. When the estimates of interest are related to phase synchronization between brain and muscles, they are related to cortico-muscular coherence or CMC. Indeed, several studies have shown that phase coupling is an effective way to describe the communication between cortex and spinal cord, where cortical oscillations have been related to different aspects of motor control (Baker et al., 1997; Jackson et al., 2002; Schoffelen et al., 2005; Baker, 2007; Bayraktaroglu et al., 2011). In particular, CMC in the beta frequency band has been related to the maintenance of specific sensorimotor states (Engel and Fries, 2010), meaning that CMC can be directly linked to motor activity. In stroke patients, it has been shown that CMC is altered in comparison to healthy populations. The observed variations are mainly described as a decrease of the CMC peak, in both acute and chronic stroke (Mima et al., 2001; von Carlowitz-Ghori et al., 2014; Guo et al., 2020). Krauth et al. (2019) investigated the effect of the ischaemic stroke on CMC estimated with EEG and EMG signals, during a wrist extension task. They compared CMC distributions to those of a group of healthy subjects. Their findings revealed that ipsilesional CMC was reduced in stroke patients in the acute phase and increased during the process of motor recovery, relating higher cortico-muscular coherence levels to better motor function, as previously reported in a case study (Zheng Y. et al., 2018). Additionally, Godlove et al. (2016) explored higher frequencies in ECoG signals (high-gamma, 76–200 Hz), finding evidence of a preserved correlation between perilesional high-gamma with the muscle synergies of the affected limb during horizontal planar movements in a human chronic stroke survivor.

Regarding alterations of CMC in response to BCI/BMI interventions employing brain signals coupled with different peripheral actuators and estimulators, since this technology aims at enhancing a biologically effective coactivation of the central and the peripheral nervous system, an increase in the CMC level of a subject undergoing a such an intervention would be expected. Not surprisingly, significantly increased EEG-EMG CMC levels between the premotor cortex and a contralateral forearm extensor muscle have been reported in chronic stroke patients in response to a short-term rehabilitative BMI intervention coupled with a robotic actuator (Belardinelli et al., 2017) or neuromuscular electrical stimulation (NMES; Mukaino et al., 2014).

It seems thus, that incorporating motor information such that a functional link between the brain and the motor output can be established is very promising to overcome the difficulty of accurately detecting the intention of movement from the ongoing brain activity. Furthermore, it has the potential to at least partly resolve previously described difficulties relating to the origin of the signals of interest (cerebral as well as muscular), because with CMC it is possible to estimate the cortical and motor relations of the desired movement, discarding muscle and brain activations caused by other unrelated processes.

That is, phase coupling measures *between sources* constitute the functional link between the brain and the peripheral nerves and muscles. Indeed, the study of cortico-muscular coherence may also constitute a tool to assess and quantify the effect of rehabilitative interventions on the patients' recovery. Additionally, this measure could potentially reveal the regions of the cortex that play a role in the process of recovery.

An important aspect to keep in mind then is the methodology employed to estimate cortico-muscular connectivity. In literature several examples exist of cortico-muscular based features for BCI data, not only to study coupling (Baker, 2007; Kristeva et al., 2007; Colamarino et al., 2021) but also attempts to deliver neurofeedback (Chowdhury and Prasad, 2019; Chowdhury et al., 2019; de Seta et al., 2022) with cortico-muscular interfaces. These works are based on *sensor-space estimates*. However, coupling estimation on sensor-space data does not enable an anatomically accurate localization of nervous system activity because each sensor records the superposed activity of all active populations (sources) in the brain and muscles. Furthermore, neurofeedback delivered with sensor-based coupling measures does not reflect any specific functional relation within the brain or between brain and muscles.

These limitations could potentially be improved with recently developed methods that allow the maximization of CMC and the extraction of maximally coupled brain and muscle sources (Bayraktaroglu et al., 2011; Vidaurre et al., 2019b). One of them has been applied in a test study to provide neurofeedback with promising results (von Carlowitz-Ghori et al., 2014).

Concluding, cortico-muscular interfaces that estimate *source related* CMC might have a greater potential for reinforcing efferent pathways from cortical motor structures to the targeted paralyzed muscles and have therefore been suggested as a promising approach for stroke motor rehabilitation (Irastorza-Landa et al., 2022). Furthermore, despite the promising preliminary results presented in this section, further work evaluating the changes in CMC levels in larger stroke patient cohorts enrolled in NI related therapy is needed to demonstrate a direct relationship with gained motor functions, hence, more controlled clinical trials are needed to test neuro-muscular interfaces in stroke survivors.

## 5. Discussion

This manuscript presented an overview of neural interfaces in stroke motor rehabilitation, highlighting the potential of this tool to reconnect brain and muscles. As seen before, motor control of the paretic limb can be improved using neuro-controlled systems.

It is hypothesized that Hebbian learning promotes motor learning, and thus matching functional efferent and afferent information in in terms of meaning and time is crucial. These variables are influenced by different aspects that have been presented in this review: the methodology employed to acquire, condition and process neural signals. Also, we saw that the evaluation of the patient's state and their involvement in the therapy are key for the success of the rehabilitative intervention.

This review also emphasizes that the success of rehabilitative NIs mainly depends on the contingency between the desired motor task and the feedback received by the patient. Thus, the performance of NIs for motor rehabilitation of stroke survivors, critically depends on the ability of the researchers to overcome the technical challenges of these systems to improve feedback contingency. In this work, we described methodological aspects that should be considered in the design of these NIs and they often revolve around increasing their accuracy and efficacy. Because brain signals are noisy and non-stationary, the automatic identification, rejection and/or correction of artifactual signals poses a great challenge for researchers and engineers. Although several recent advances exist (see Section 3.1) to date there is no method able to completely reject non-linear artifacts. Fortunately, some recent interesting approaches toward this goal were recently published (see e.g., Chen et al., 2022).

Besides, the need of data to recalibrate BCI systems in each session is also a disadvantage of current interfaces. The physical and cognitive state of the patients, who cannot undergo long and exhausting rehabilitation sessions, requires neuro-feedback systems that can be speedily prepared. The session-to-session transfer of the models used to process data has been investigated to ameliorate setup periods (see e.g., Arvaneh et al., 2013; Azab et al., 2019). For example, adding data from the same session has been shown to improve performance in healthy population (Vidaurre et al., 2007a) as well as in patients (López-Larraz et al., 2018b). Furthermore, co-adaptation also seems like a promising a approach that can incorporate past data and current signals to improve performance (Nierhaus et al., 2021; Zhang et al., 2022). Nevertheless, care should be taken on how adapt to the signals of interest to boost the rehabilitation outcome.

The technical consideration that is continuously considered in this review from different angles is the improvement of the contingency between cortical activations and feedback based on the decoding result. A correct feedback is thought to be key to stimulate pertinent brain networks to restore motor function. However, as seen throughout the text, the lack of specificity of brain signals might seriously affect the decoding performance of neural interfaces to restore movement and consequently the quality of the feedback provided, therefore a multimodal approach might be the most successful option.

However, the strategy followed to control the interface, that is, how to combine signals to obtain one specific command, is also a challenging technical requirement because it should mainly reflect the intention of the specific movement that should be restored. Different approaches were discussed in Section 4. From those, the estimation of cortico-muscular coherence at the source level vs. electrode domain seems very promising (von Carlowitz-Ghori et al., 2014). We believe that the use of features based on the communication between brain and muscle sources related to the motor task of interest are key for future NI systems for motor rehabilitation. These features should be reliable and estimated in sufficiently short windows to ensure online control (de Seta et al., 2022). Additionally, an external peripheral actuator could be employed to ensure natural and functional sensorimotor feedback. In short, efforts must be done to ensure high control stemming from the voluntary movement to reinforce the restoring of functional neural recruitment. Neurophysiological data recorded in clinical studies appplying neuro-controlled paradigms offers a valuable tool to access and study new neurophysiological bio-targets.

Other aspects that might limit the efficiency of current interfaces are related to the state of the specific type of patient. For example, stroke survivors often suffer from loss of muscle volume and tone, but most of them retain residual muscle activity. Thus, including EMG signals in the interface would tackle muscle involvement and thus, muscle atrophy. In the same line, FES could be a valuable tool to increase muscle tone. Moreover, changes in bio-mechanics, mainly due to tendon and muscle shortening, can be a key factor limiting recovery and movement rehabilitation. Indeed, this could be an underestimated factor influencing spasticity measures (Mahmoud et al., 2023). In this context, neuro-controlled body actuators might be the key to open the gateway to spasticity reduction (Ramos-Murguialday et al., 2013).

Not only spasticity is present after stroke, but also maladaptive muscle synergies or patterns may prevent coordinated multi-joint movements to occur. Therefore, EMG-based control of body actuators might be necessary to re-adapt or re-learn appropriate muscle control (Cheung et al., 2018). However, in case of distal activity only, the top-down volley might not coincide correctly with distal activity, especially if spasticity is a result of changes at subcortical and also spinal neural structures. Therefore and as previously discussed, properly assessed hybrid brain-muscle control of the body actuator, for example with the help of source phase coupling estimators (Vidaurre et al., 2019b), might assist the re-learning process in a more efficient manner.

Finally, longitudinal MRI studies indicated that clinical improvements were associated with an increased activation of the ipsilesional hemisphere (Caria et al., 2011). In light of this, it could be expected that interventions that aim to reverse these alternative motor control mechanisms and enforce the primary control mechanism would have an effect on spasticity expression. The evident effectiveness of some recent interventions lends support to this argument. Indeed, it has been reported that successful therapeutic NI-based intervention produces restoration of motor function mediated by re-lateralization of motor cortical activation (Ward et al., 2003; Schlaug et al., 2008; Ramos-Murguialday et al., 2013; Song et al., 2014; Ray et al., 2020).

Regarding feedback modalities, it is important to consider that although sensory function is compromised in stroke patients, most of them present sufficient afferent input to the brain to be able to learn and use proprioception (Ramos-Murguialday et al., 2013) or sensory electrical stimulation (Tu-Chan et al., 2017) as feedback. Compromised sensory systems need to be assessed, as well as behavioral, neurophysiological and clinical status, since patients' triage is key for therapy individualization and success.

Moreover, NI designed as serious games and immersive systems could help motivate patients. Having extra sensors to optimize their performance (measuring cognitive and physical fatigue and attention) can help adjust intervention difficulty and intensity, thereby easing adoption, acceptance, and engagement.

## 6. Conclusion

This work presented the main methodological and neurophysiological considerations on the design of neural interfaces for rehabilitative purposes. While most of designs are proposed and tested in healthy subjects, translating these findings into a clinical population requires some important adjustments.

Advanced methods are being developed to keep improving the performance of BCI/BMIs. Maybe, the primary target when designing rehabilitative systems should be the careful assessment of a direct link between the “correct/functional” neural activity and the feedback. Higher performances can result in an unsuccessful intervention if feedback provided relies on unrelated activity (i.e., the subject has learned to control the interface via compensatory movements or biased signals). Because of this, features directly linked with neurophysiological events are highly recommendable for rehabilitative purposes.

When designing rehabilitative neural interfaces for stroke, one should consider other several aspects: the neurophysiological state of the patients, residual neural signals conveying motor-related information, preserved and maladapted motor functions, etc.

Customized therapies as well as standardized approaches are essential for building evidence and reach consensus in research. Reporting complementary analyses of neurophysological changes as an effect of rehabilitative interventions in addition to standard (subjective) clinical scales is of paramount importance to understand the plasticity mechanism happening at central and peripheral levels.

Novel neural interfaces should be designed to use and reinforce neurophysiological features that have been found to correlate with motor function recovery. Furthermore, there is a clear lack of studies longitudinally investigating the chronic phase and the development of the sensorimotor system in stroke, including plasticity and spasticity mechanisms conditioning the rehabilitation potential of neural interfaces. Special attention should be paid to the design of scientifically based rehabilitation tasks (movements) that can be executed by neural controlled body actuators, which can leverage and generalize NIs rehabilitative effects.

## Data availability statement

The original contributions presented in the study are included in the article/supplementary material, further inquiries can be directed to the corresponding author.

## Author contributions

AS-S, AI-D, AR, WM, CB, FH, IO-I, EL-L, HL, and AR-M drafted sections of the text. NI-L and CV prepared the manuscript. AR-M wrote sections of the manuscript and revised the text. All authors contributed to the article and approved the submitted version.
